# Appropriation of Mobile Health for Diabetes Self-Management: Lessons From Two Qualitative Studies

**DOI:** 10.2196/10271

**Published:** 2019-03-29

**Authors:** Constanze Rossmann, Claudia Riesmeyer, Nicola Brew-Sam, Veronika Karnowski, Sven Joeckel, Arul Chib, Rich Ling

**Affiliations:** 1 Department of Media and Communication Science University of Erfurt Erfurt Germany; 2 Ludwig Maximilian University of Munich Munich Germany; 3 Wee Kim Wee School of Communication and Information Nanyang Technological University Singapore Singapore

**Keywords:** diabetes, Germany, mHealth, mobile phone, self-management, Singapore

## Abstract

**Background:**

To achieve clarity on mobile health’s (mHealth’s) potential in the diabetes context, it is necessary to understand potential users’ needs and expectations, as well as the factors determining their mHealth use. Recently, a few studies have examined the user perspective in the mHealth context, but their explanatory value is constrained because of their limitation to adoption factors.

**Objective:**

This paper uses the mobile phone appropriation model to examine how individuals with type 1 or type 2 diabetes integrate mobile technology into their everyday self-management. The study advances the field beyond mere usage metrics or the simple dichotomy of adoption versus rejection.

**Methods:**

Data were gathered in 2 qualitative studies in Singapore and Germany, with 21 and 16 respondents, respectively. Conducting semistructured interviews, we asked respondents about their explicit use of diabetes-related apps, their general use of varied mobile technologies to manage their disease, and their daily practices of self-management.

**Results:**

The analysis revealed that although some individuals with diabetes used dedicated diabetes apps, most used tools across the entire mobile-media spectrum, including lifestyle and messaging apps, traditional health information websites and forums. The material indicated general barriers to usage, including financial, technical, and temporal restrictions.

**Conclusions:**

In sum, we find that use patterns differ regarding users’ evaluations, expectancies, and appropriation styles, which might explain the inconclusive picture of effects studies in the diabetes mHealth context.

## Introduction

### Background

Diabetes has increasingly become a major burden for industrialized societies, with rising health care costs and mortality rates pressuring governments globally to address this problem [1,2]. These governments have finally started to recognize the problem’s seriousness. Singapore, the first country that we examined, has the second-highest diabetes prevalence rate after the United States [3], and it launched a war on diabetes in 2016. Germany, the second country that we examined, ranks third for diabetes-related health expenditures worldwide [2,4].

With the rapid growth and ubiquitous availability of mobile phones, mobile health (mHealth), that is, “the use of mobile communications for health information and services” [5], can potentially contribute to improving health promotion, disease prevention, and disease self-management [6-10]. In the diabetes context, functions such as messaging and chatting with health care providers, connections to external devices (eg, heart-rate measurement and monitoring of blood glucose or blood pressure), and support of medication, as well as tracking physical activity or nutrition behavior via mobile apps are discussed [11,12].

Up to this point, research has mainly focused on mHealth’s effects, indicating not only promising results overall [13,14] but also contradictory empirical evidence [15-19]. Even a recent systematic review concluded that research is still too heterogeneous and somewhat too low in methodological quality to “provide reliable evidence of effects for stakeholders” [12]. To gain a clearer picture of mHealth’s potential in the diabetes context, it is necessary to understand potential users’ needs and expectations, as well as the factors determining their mHealth use. In recent years, more and more studies have dealt with the user perspective in the mHealth context [20-39]. However, most of these studies have fallen short in their ability to explain more than just adoption factors.

Our goal is to describe different patterns of everyday life integration that go beyond mere usage metrics or the simple dichotomy of adoption versus rejection [40]. To achieve this goal, we draw on the mobile phone appropriation (MPA) model [41] as our theoretical frame. Empirically, we conducted semistructured interviews with diabetes patients in Singapore (study 1) and Germany (study 2). Study 1 identified relevant functional, normative, symbolic, and restriction evaluations tied to diabetes app use, whereas study 2 complemented these evaluations for mHealth appropriation and identified supplemental patterns of evaluation, use, and meta-communication.

### Mobile Health and Diabetes Self-Management

Current extant research on mobile devices’ and services’ effects on health outcomes often focuses on SMS text message interventions [14,42]. Increasingly, studies have concentrated on smartphone apps’ effects on diabetes self-management [12,13,15]. However, their results are diverse, ranging from positive effects on diabetes outcomes, for example, hemoglobin A_1c_ reduction [43], to limited or no effects [44,45]. Moreover, recent systematic reviews are not consistent but reveal positive effects overall [13,14]. Studies have increasingly tried to specify effects by varying message design and tailoring messages to users’ needs [46-49], with promising results. However, also in this context, a clear picture cannot be drawn so far. This may be explained, at least partly, by an overly simplistic idea of use and effects. Using apps or receiving certain messages does not tell us how users interact with apps and interpret their functions [18].

Thus, the need exists for a better understanding of actual everyday-life use and mobile devices’ integration into diabetes self-management. To increase our knowledge about mHealth’s role in the diabetes context, we need to ask how, why, and for what purpose patients use mobile devices for diabetes self-management and which motives, perceptions, and expectations drive their use.

Primarily, the answers to these questions require that we define what we mean by *use*. In extant literature, *use* has been used quite heterogeneously, describing all kinds of processes and subprocesses in decision and implementation phases, as defined by Rogers’ [50] innovation-decision process. These 2 stages help distinguish between 2 broad areas of use: first, Rogers describes the decision stage (adoption), in which the overall question of use versus nonuse (ie, adoption vs nonadoption) is tackled. The second phase of implementation (appropriation) deals with the question of everyday-life integration and actual use patterns [50,51].

In recent years, more and more studies have focused on mHealth adoption, drawing mostly on the technology-acceptance model (TAM) [24,25,27,33,37,51,52] or its successor, as well as the unified theory of acceptance and use of technology (UTAUT) [30,35,38,39,53], to explain influences on the adoption decision. However, only a few studies have considered an implementation or appropriation perspective on mHealth use, mostly focusing on continued use in contrast to the single-adoption decision. These studies fail to consider the multifaceted patterns of everyday-life integration [21,26,37].

In the diabetes context, most studies that concentrate on the implementation process are rather descriptive in nature, examining frequency of use or expectations regarding diabetes apps [20,22,29].

### Appropriation of Mobile Media

**Figure 1:**
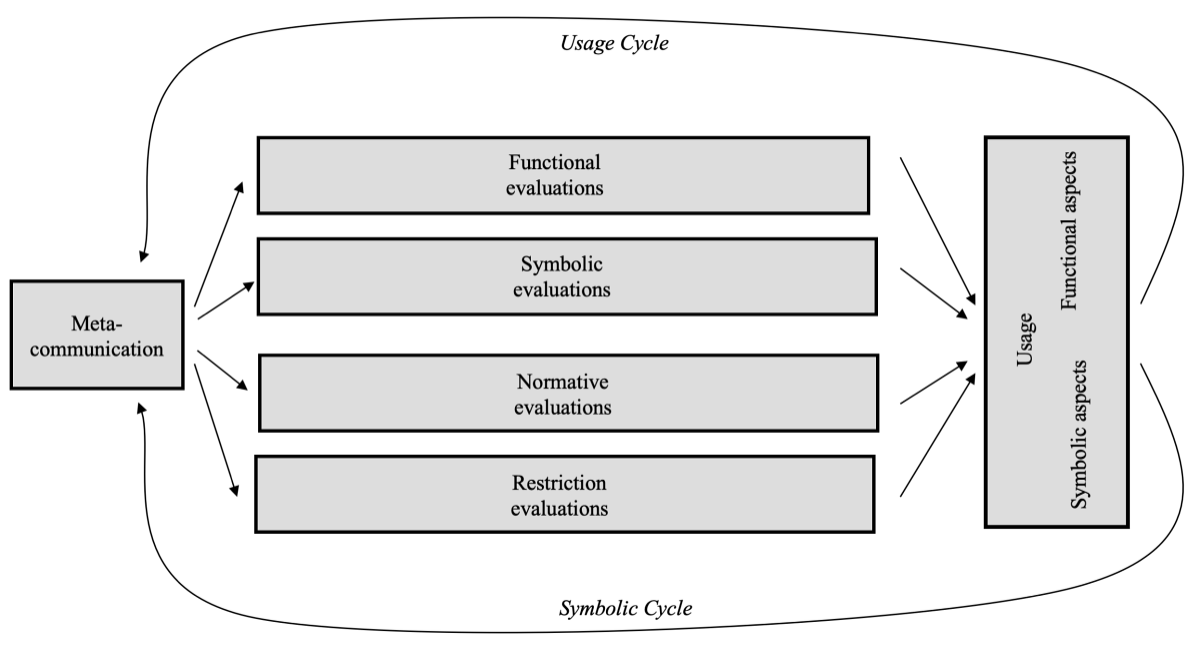
The mobile phone appropriation model (simplified).

The MPA model [41] provides a theoretical framework with which to analyze the appropriation of mobile media as a process (Figure 1), not only in general but also in specific contexts [54]. The model integrates concepts of technology adoption, for example, diffusion of innovations [50], theory of planned behavior [55], TAM [52], and UTAUT [53], with conceptualizations of the actual use and implementation of technological innovations into users’ everyday lives. On the basis of cultural studies [56,57] and the domestication approach [58], Wirth et al [41] term this process *appropriation*, emphasizing users’ active co-construction of meaning, thereby overcoming the *binary logic of adoption* [40].

The MPA model conceptualizes appropriation as a creative and active process, resulting in various use patterns by individual mobile-media users. Consequently, behavior is differentiated in sub-constructs in the MPA model, comprising symbolic and functional aspects. The functional aspects represent the variety of uses, for example, highlighted in research on the basis of the uses and gratifications approach. By adding symbolic aspects such as prestige, the MPA captures the concept and extent of observability [50], making the choice and use of mobile media a continuous statement about oneself in public [59]. Functional, symbolic, normative, and restriction evaluations influence these use dimensions. Functional and symbolic evaluations represent users’ beliefs about the functional and symbolic aspects of their future mobile-media behaviors. Normative evaluations refer to their beliefs and judgments about social norms related to their future behavior. Restriction evaluations—comprising financial, technical, cognitive, and temporal factors—represent users’ beliefs about constraints hindering their future mobile-media behaviors. Restriction evaluations find resonance with the information communication technologies for the health care model used in mHealth studies [60,61], which propose economic, technological, infrastructural, and sociocultural barriers.

In addition, the MPA model integrates meta-communication, that is, the impact of communication on communication technologies. As users communicate about their respective uses of mobile media and observe others’ behaviors, this meta-communication influences their future behaviors. Consequently, the MPA model is conceptualized as a cycle, with the appropriation being a constantly renewed process. Functional and symbolic mobile uses are not only the results of behavioral, normative, or restriction beliefs but they also become the basis of those beliefs [62].

So far, the MPA model has been adapted successfully to the mHealth context in 1 study examining patterns in nutrition-app appropriation [63]. On the basis of a Web-based survey of nutrition app users, the study identified 4 distinct appropriation types: supported, indifferent, health-conscious, and socializer. These types differed mainly regarding (1) the support they received from their social peers for their app use, (2) their personal attachment to their app use, and (3) app use for socializing (and competition). Thus, we see great potential in using the MPA model as a theoretical framework to gain insight into the appropriation processes of mobile services and devices for diabetes self-management and ask these research questions (RQs):

RQ1: Which specific functional, symbolic, normative, and restriction evaluations are relevant in the context of mHealth appropriation for diabetes self-management?

study 1

RQ2: What role does meta-communication play in mHealth appropriation for diabetes self-management?

study 1

RQ3: Which patterns of mHealth appropriation can be found in the context of diabetes self-management?

study 2

To answer these questions, we carried out 2 related yet independent studies that focused on (1) different aspects of the appropriation process and (2) 2 different cases (Singapore and Germany). Study 1 lays the foundation for investigating app appropriation, and study 2 expands this notion by looking at the broader picture of mHealth for diabetes self-management.

## Methods

### Study 1 Method

Study 1 (Singapore) focused on the appropriation and use of diabetes-specific apps for self-management (*diabetes apps*). These apps are designed specifically to support diabetes self-management, including tools for blood sugar monitoring and direct feedback (diabetes log books, eg, *Glyco App*), diabetes information (eg, *MySugr Academy*), or food databases (for counting carbohydrates to adjust insulin, eg, *the Singaporean Health Promotion Board’s food database*). We carried out 21 semistructured, face-to-face interviews [64,65] (approximately 1 hour each, in English) with Singaporean type 1 and type 2 diabetes (and prediabetes) patients between December 2015 and September 2016. Singaporean diabetes-support groups (Diabetes and Diabetic Society of Singapore) were used to contact diabetes patients. The participants were asked to choose the interview locations to make them feel as comfortable as possible throughout the interviews.

The interviewees were recruited in such a way that the greatest possible variance in demographic characteristics—gender, age, diabetes type, period since diagnosis, and form of therapy—could be covered [65,66]. The prerequisites for participation were the following: having an existing diabetic condition and being a Singaporean resident. Using such a broad cross-section of participants was undoubtedly a challenge, but only in this way was it possible for us to grasp the combination of different characteristics as widely as possible to gain insight into the use of mobile media in the diabetes context. Focusing on 1 or 2 characteristics (eg, only 1 diabetes type, 1 age group, or 1 type of treatment) would have restricted this view. Table 1 provides an overview of sample characteristics, and Table 2 provides additional information on participants. Moreover, it should be noted that 8 participants suffered from other diseases in addition to diabetes (eg, heart conditions, high blood pressure, high cholesterol, hypothyroidism, and/or breast cancer), 17 participants received diabetes education at some point, and 15 were part of a diabetes support group.

Both in the construction of the interview guide (see Multimedia Appendices 1 and 2) and in the analysis of the interviews, we followed a theory-driven approach, which differs from classic grounded theory or hermeneutics [67]. The interview guide was based on the MPA model and assessed the diabetes context, general daily diabetes self-management, and the use of Web-based (mobile) devices as part of diabetes self-care. It included 30 flexible questions, that is, if answers to a specific question had been provided before, the question was omitted. All interviews were transcribed. The resulting transcripts each totaled 5000 to 10,000 words and were analyzed following a theory-driven approach on the basis of the research questions and the MPA model [41]. The data analysis followed a thematic-analysis approach as described by Braun and Clarke (2006), who define *thematic analysis* as “a method for identifying, analyzing, and reporting patterns (themes) within data” (p. 79), and it can be called “theoretical thematic analysis” because the themes are derived (at least partly) from the theoretical background, in contrast to an inductive approach [68]. The analysis was based on the categories’ *functional*, *normative*, *symbolic*, and *restriction evaluations* as described in the MPA model, and it used the interview data to build themes around these theoretical concepts to identify commonalities and differences, as well as understand the appropriation of diabetes-specific apps for self-management in detail. If important interview extracts did not fit into existing categories on the basis of the MPA model, new subcategories were added. The procedure of creating categories and themes was dynamic and constantly adapted on the basis of the interview content. An institutional review board approval was received by NTU Singapore for the face-to-face interviews.

### Study 2 Method

On the basis of the results obtained from study 1, study 2 asked for patterns of mHealth appropriation that can be found in the context of diabetes self-management. From June 2017 to August 2017, we conducted 16 semistructured interviews with German individuals with diabetes [64,65]. They were recruited through a purposive-sampling approach to cover a variance in the characteristics of age, diabetes type, period since diagnosis, and treatment [69-71]. We recruited interviewees via doctors in private practices and hospitals in Munich and Jena. Tables 3 and 4 provide more information about the sample.

The interview guide was based on our theoretical assumptions [41], study 1’s results, and the interview guide developed for study 1. We adjusted it for language, as well as a broader understanding of mHealth use beyond specific diabetes apps. We asked the participants about (1) their smartphone use, (2) their knowledge about mobile media, (3) their attitude toward the use of mobile media in the context of diabetes, and (4) their self-management of their disease with the support of mobile media. The interview guide covered 21 questions.

**Table 1:** Singaporean sample characteristics (N=21).

Characteristic		n (%)
**Gender**		
	Male	11 (52)
	Female	10 (48)
**Diabetes type**		
	Type 1	9 (43)
	Type 2	11 (52)
	Other (prediabetes)	1 (5)

**Table 2:** Singaporean sample.

Generic name^a^	Diabetes type	Age (years)	Gender	Years since diagnosis	Treatment
Kang	Prediabetes	67	Male	8	No medication
Adit	1	22	Male	10	Insulin (injection)
Cheng	1	23	Male	9	Insulin (injection)
Jie	1	64	Male	37	Insulin (injection) and Metformin or similar (oral)
Kaiyan	1	42	Female	36	Insulin (injection)
Navin	1	58	Male	38	Insulin (injection, pump)
Pang	1	19	Male	7	Insulin (injection)
Sona	1	20	Female	17	Insulin (injection)
Shi Hui	1	35	Female	28	Insulin (injection)
Xiu Wen	1	57	Male	32	Insulin (injection)
Bharat	2	66	Male	34	Insulin (injection)
Ching	2	64	Female	10	Metformin or similar (oral)
Deng Li	2	68	Female	4	Metformin or similar (oral)
Ei Tek	2	60	Male	31	Insulin (injection) and Metformin or similar (oral)
Gu Fang	2	29	Female	20	Insulin (injection, pump) and Metformin or similar (oral)
Henna	2	60	Female	24	Insulin (injection) and Metformin or similar (oral)
Li Ting	2	49	Female	9	Insulin (injection) and Metformin or similar (oral)
Ming	2	—^b^	Male	12	Metformin or similar (oral)
Rei Hong	2	61	Male	9	Metformin or similar (oral)
Xin Qi	2	56	Female	7	Metformin or similar (oral)
Zhen Wei	2	47	Female	18	Metformin or similar (oral)

^a^The transcripts were anonymized, and participants were given a generic name that matches with the in-text quotations.

^b^Age unknown.

The interviews lasted between 30 min and 60 min each, and they were audiotaped and transcribed into written form. The transcripts covered between 5000 and 10,200 words per interview. Our analysis was based on our paper’s theoretical concept (MPA model) and study 1’s results. We analyzed the interviews following the data-analysis process suggested by Creswell [71]. We read all transcripts, marked relevant passages, and abstracted them until we found the dimensions of mediated communication, diabetes self-management, and social prestige and control, ordering the presentation of the results. All responses were allocated to these 3 dimensions, and we identified similarities and differences among participants’ usage patterns and linked study 2’s results to the MPA model. The Research Support Office of University Hospital Jena checked the interview guide and approved it. Information on sample characteristics can be found in Table 3, and the participants are described in Table 4.

Type 1 patients used the *Freestyle Libre* device by Abbot (n=5), Continuous Glucose Monitoring by Dexcom (n=2), or *Accu-Chek* by Avia with *Contour-App* (n=1) to test blood sugar levels. Only 1 patient relied only on test strips. Type 2 patients did not self-test blood sugar levels. Participants used IOS (n=6) or Android (n=10) devices. Only Andreas, aged 71 years and Linda, aged 42 years, used smartphones older than 5 years. In total, 9 participants used smartphones, which they had for less than 2 years.

**Table 3:** German sample characteristics (N=16).

Characteristic		n (%)
**Gender**		
	Male	7 (44)
	Female	9 (56)
**Diabetes type**		
	Type 1	11 (69)
	Type 2	4 (25)
	Other (prediabetes)	1 (6)

**Table 4:** German sample.

Generic name	Diabetes type	Age (years)	Gender	Years since diagnosis	Treatment
Fiona	Prediabetes	29	Female	0	Unknown
Andreas	1	71	Male	18	Insulin (injection, pump)
Ben	1	64	Male	49	Insulin (injection, pump)
Conrad	1	31	Male	19	Insulin (injection)
Daniela	1	32	Female	22	Insulin (injection)
Emma	1	45	Female	30	Insulin (injection, pump)
Gerd	1	32	Male	20	Insulin (injection, pump)
Katja	1	43	Female	30	Insulin (injection)
Linda	1	42	Female	27	Insulin (injection, pump)
Olga	1	52	Female	35	Insulin (injection, pump)
Petra	1	25	Female	20	Insulin (injection)
Stefan	1	25	Male	9	Insulin (injection)
Jessica	2	56	Female	10	Metformin or similar (oral)
Marc	2	64	Male	0	Unknown
Norbert	2	58	Male	12	Insulin (injection, oral)
Ramona	2	43	Female	0	Metformin or similar (oral)

## Results

### Study 1 Results

Our first research question inquired about which specific functional, symbolic, normative, and restriction evaluations were relevant in the context of mHealth appropriation for diabetes self-management. Patients generally differed in their appropriation of apps designed for diabetes, with their use ranging from no previous use and no knowledge about existing diabetes self-management apps to infrequent and short-term app use as well as to long-term app use. The length of app use varied significantly, from a few days to several months or even years. Almost all type 1 diabetes patients reported using diabetes apps, whereas just a few type 2 patients used diabetes apps for their self-management. In addition, the interviews revealed that high-risk diabetic patients, that is, those with critical conditions or insufficient self-management, did not use diabetes apps (eg, Li Ting, aged 49 years; Ming, age unknown; Rei Hong, aged 61 years; Zhen Wei, aged 47 years).

#### Functional Use and Handling of Diabetes Apps

In terms of functional evaluations, the participants mainly mentioned diabetes monitoring and nutrition information. Diabetes monitoring almost exclusively referred to the use of blood glucose log books with a diary function to track blood sugar fluctuations (Dose Adjusting For Normal Eating app, *mySugr*, *Glooko*, Health Promotion Board *HPB* app, and *Diabetes M*). Nutrition information was related to the use of diabetes-database apps for gathering information about food content:

Another app I think will be useful is an app that is able to calculate for you the calories that you’re going to be eating (...) So, I just have to enter [the food type] into the app and then it will work out for me how much carbohydrate.

Kang, aged 67 years

#### Diabetes Monitoring

For some, log-book apps replaced paper-and-pencil blood sugar logs by typing blood sugar results from the blood glucose meter into the app (eg, Bharat, aged 66 years; Cheng, aged 23 years; Kaiyan, aged 42 years; Shi Hui, aged 35 years; Navin, aged 58 years). Some individuals with diabetes used log books that were automatically synchronized with glucose meters via Bluetooth (Henna, aged 60 years; Kaiyan, aged 42 years). The preference clearly leaned toward automated systems to facilitate glucose monitoring and avoided time-consuming monitoring processes (Sona, aged 20 years; Adit, aged 22 years). In addition, participants viewed log-book apps’ automated data analysis to be useful, especially the improved sharing of blood sugar levels with health care professionals (Henna, aged 60 years; Shi Hui, aged 35 years). Some apps allowed data sharing with medical staff via clouds, whereas others used email (PDFs or Excel spreadsheets). However, cloud-based data sharing was linked to a minority of app users in the sample (Gu Fang, aged 29 years; Sona, aged 20 years), which can be partly explained by the fact that some participants expressed data-protection concerns. Xiu Wen, aged 57 years, explained in the following manner:

If you put medical information in the cloud, then this becomes a...data-privacy issue.

Moreover, according to interview participants, Singaporean physicians were rather reluctant to recommend self-management diabetes apps that the government did not support—for a list of government-supported apps, please refer to the Singaporean Ministry of Health website [72]. Kang, aged 67 years, noted the following:

Our doctors and staff...they have to be careful...If, for example, the doctor says, “Oh. Try this app.” ...then if something goes wrong, they will publish it in the newspaper,...or they put it on Facebook. So, they don’t try and say, “Oh, maybe you should try this app.” ...They will never push it unless, if it’s through the government.

Participants who reported using cloud services also used other mobile devices (such as step- and sleep-trackers, or glucose meters) and additional apps connected to diabetes apps (Henna, aged 60 years; Kaiyan, aged 42 years; Shi Hui, aged 35 years), thereby making broader use of their whole mobile-media ecosystem. Both type 1 and type 2 patients used log-book apps and reported their usefulness.

#### Nutrition Information

Mainly individuals with type 1 diabetes reported using food databases (Adit, aged 22 years; Cheng, aged 23 years; Pang, aged 19 years), likely because of type 1 patients’ greater need for food-content information to accurately adjust their insulin with food intake. Cheng, aged 23 years, explained in the following manner:

I roughly know my diet and my food, so I do the carb counting and stuff.

Pang, 19, said,

The health-promotion board...I know they have an app for that [food database]; they also have it online so...whenever aaah...let’s say I am unsure about how much carbohydrate a food has...(I) can always go look it up.

Detailed nutrition information was less relevant for type 2 patients who did not inject insulin. Apart from diabetes monitoring and nutrition information, the participants did not mention any further diabetes-specific app functions. Due to perceived limitations in diabetes-specific apps for self-management (see restriction evaluations), the participants used additional mobile devices and services for their daily self-management, including general health-information apps (eg, *WebMD*, *Health Buddy*), health and body mass index calculators, fitness apps (eg, *MyFitnessPal*), instant messengers (eg, *WhatsApp*), heart-rate monitors, and step- and sleep-trackers. Thus, on a more general level (not limited to diabetes apps), functional evaluations of health information and communication can be added to monitoring and nutrition information.

#### Symbolic Evaluations

Symbolic evaluations, which have been proven to play a role in the context of mobile phone appropriation in general, were not mentioned in the interviews.

#### Normative Evaluations

The influence of normative evaluations depends on patients’ relationships with their doctors, and it is mainly seen with dependent patients who prefer to follow their physicians’ instructions closely (Ei Tek, aged 60 years; Li Ting, aged 49 years; Ming, age unknown; Rei Hong, aged 61 years). Mostly, these patients did not use diabetes apps, and they were either skeptical of them or had no knowledge on how to use diabetes apps (Ei Tek, aged 60 years; Li Ting, aged 49 years; Ming, age unknown; Rei Hong, aged 61 years). These attitudes may reflect the prevailing sociocultural norms or perceptions of their personal physicians’ views on apps.

#### Restriction Evaluations

Respondents often mentioned barriers to diabetes-app use and reasons for stopping app use. The reported evaluations mirror the 4 restriction categories, which the MPA model proposed: financial, temporal, cognitive, and technical. Financial barriers are mainly related to unwillingness to pay for diabetes apps. Shui Hui, aged 35 years, stated the following:

Not all patients would be willing to pay.

Temporal restrictions are related to the time required to use app log books, for example, for monitoring blood sugar levels (Xiu Wen, aged 57 years). A lack of knowledge about app availability and use was reported as a cognitive barrier (Xiu Wen, aged 57 years). Technical barriers included technical failures, with some diabetes apps frequently *crashing* (Cheng, aged 23 years), resulting in a lack of reliability. In addition, 1 diabetes patient reported technical incompatibilities between diabetes apps and blood glucose meters (Gu Fang, aged 29 years). Overall, diabetes patients stressed that they did not perceive the apps to be a solution for diabetes-related challenges in general but rather as additional tools for patients with diabetes who generally are motivated and have enough knowledge about self-management:

How motivated is the patient...somebody who's...very energetic,...it's interesting, you know, something that's new to them, they'll do it.

Xiu Wen, aged 57 years

#### Meta-Communication

Our second research question inquired into what role meta-communication played in the appropriation of mHealth for diabetes self-management. Communication on the use of diabetes apps for self-management can be divided into communication with other diabetic patients or peers and communication with health care providers. Patients mentioned using Web-based chats, for example, *WhatsApp* (Cheng, aged 23 years; Ei Tek, aged 60 years), to discuss topics around diabetes management with other diabetic patients. Moreover, 1 diabetes patient participating in patient support groups mentioned chats as being relevant for information exchange and organization (Bharat, aged 66 years).

Meta-communication with health care providers played a relatively minor role. As mentioned, participants reported that their doctors *never* (Li Ting, aged 49 years) mentioned or recommended diabetes apps and rarely introduced new technological options to them (Ching Ching, aged 64 years), possibly being reluctant to recommend technology that the government has not tested and approved officially (Kang, aged 67 years).

### Conclusions for Study 1

In summary, the interviews in Singapore revealed that evaluations of diabetes apps’ usefulness for self-management differed largely in the sample and across patient types (eg, motivated vs unmotivated patients). Some participants found diabetes apps to be useful for daily self-management, used 1 diabetes app over a longer period, switched among different apps, or used various apps concurrently. Other participants did not perceive diabetes apps to be useful and stopped using them after the first trial or did not try apps for self-management at all. Although most participants tested or used diabetes apps, our results show that other mobile services and devices that are not necessarily diabetes-specific (eg, fitness trackers, dietary apps, and instant messaging) are used in addition to or instead of diabetes apps. Thus, study 1 indicates that Singaporean individuals with diabetes do not use diabetes apps exclusively but rather make use of the broader mobile-media ecosystem to manage their disease. Therefore, in the next study, we broadened our focus beyond specific diabetes apps to other tools in the mobile ecosystem.

### Study 2 Results

We identified individual evaluations and, unlike study 1, further synthesized them into distinct appropriation patterns following the MPA model’s [41] logic to answer our third research question. In total, 3 overarching dimensions of mHealth appropriation for diabetes self-management emerged: mediated communication comprising the functions of information gathering and social connectedness, diabetes self-management, including self-treatment, testing, and lifestyle management, and social prestige and control.

#### Mediated Communication

Diabetes patients, regardless of their diabetes type, used digital information to learn more about their disease, including *Google* (Linda, aged 42 years; Stefan, aged 25 years; Jessica, aged 56 years), *Wikipedia* (Marc, aged 64 years), or broad-spectrum websites to gather information about new diabetes developments (Ramona, aged 43 years; Marc, aged 64 years). Type 1 patients indicated gathering critical information on new technologies such as insulin pumps from corporate websites (Andreas, aged 71 years) or online forums (Emma, aged 45 years). Most of this information gathering was restricted to traditional forms of Electronic health (eHealth), mainly via the computer (Ramona, aged 43 years) and, to a lesser extent, via mobile devices. Younger participants used mobile devices (Petra, aged 25 years), whereas older patients or those with eye problems, regardless of diabetes type, complained about mobile devices’ *small screen size* (Katja, aged 43 years), which can be interpreted as a technological restriction within mobile devices for people with varying physical conditions [73]:

You always have to put on your glasses on your mobile phone.

Andreas, aged 71 years

Gerd, aged 32 years, who followed a diabetic coaching list and self-help groups, mentioned *WhatsApp* as an additional information source beyond traditional Web-based searches (and not just as a communication tool).

With respect to social connectedness, other nonmediated forms of communication emerged as salient tools. Respondents talked face-to-face *to a neighbor* about diabetes (Norbert, aged 58 years) or with *my colleague’s daughter* (Linda, aged 42 years), who also suffered from diabetes. These conversations continued on the Web, partly enabled by mobile devices. In general, the participants described a give-and-take approach to exchanging experiences, both asking for and providing information, for example, concerning technological developments. Some respondents also participated in discussions on the Web on costs, insurance, or specific self-management apps (Conrad, aged 31 years; Fiona, aged 29 years; Olga, aged 52 years). Others were more critical and described some forums as *hysterical*, highlighting that information gathered there needed to be put into perspective by their physicians. Jessica, aged 56 years, stated the following:

Often, there is a discussion in the forum, then a doctor intervenes and writes, “So, I am a doctor, and I can now say this and that.” And then he corrects some things when someone has written something totally bad.

*WhatsApp* played an important role, with participants taking part in certain thematic, for example, food-focused, groups (Jessica, aged 56 years; Ben, aged 64 years). Gerd, aged 32 years, described an interesting case of social connectedness and exchange in mHealth by posting his blood glucose levels on the Web for his remote diabetes counselor to access. When far apart, the counselor used *WhatsApp* to contact Gerd immediately if some values were out of bounds. Nevertheless, such instrumental use of social media apps was a rare occurrence in the interviews as most patients only used such apps to exchange information with peers or other patients and not to expand doctor-patient communication.

Moreover, 1 restriction observed in the use of mediated communication for social connectedness and exchange, both mobile and static, was that information on the internet was not always considered trustworthy (Daniela, aged 32 years). On a more symbolic dimension, those in need of exchange were deemed incapable of self-management, or as Daniela, aged 32 years, stated, *wimpy*.

#### Diabetes Self-Management

For diabetes self-management, we saw a rather clear-cut distinction between type 1 and type 2 patients—a pattern also seen in study 1’s results with respect to app use. Although self-treatment and self-testing were more relevant for type 1 patients, lifestyle was a relevant subdimension for patients of both types.

Self-treatment and self-testing mostly related to the use of smartphones for measuring and tracking blood glucose levels and/or for using diabetes-management apps, such as *My Sugar*. For diabetes self-management, the *Freestyle Libre* app played a crucial role, giving type 1 patients more control and autonomy on a symbolic level. Stefan, aged 25 years, thinks these apps are *cool*. However, we also observed several restrictions. Even though devices like *Freestyle Libre* and their connectivity with smartphones gave users more autonomy, they traded this freedom for a reliance on their smartphones:

I am depending on battery life.

Fiona, aged 29 years; Gerd, aged 32 years

IOS (Apple operating system) users like Petra, aged 25 years, could not use the *Freestyle Libre* app as it was incompatible with IOS (at the time of the study). Linda, aged 42 years, who used 1 of the oldest phones in the sample, simply could not use any measurement apps, as her phone did not support them. Olga, aged 52 years, used a Continuous Glucose Monitoring (CGM) sensor and the *Stealwise App* on a Sony Z5. She mentioned financial constraints as *health costs a lot of money*. Ben, aged 64 years, using a new iPhone and CGM sensors, stated the following:

If I cannot use the device, it goes along with not being able to use the app.

As previously noted, the use of smartphones as platforms for self-testing apps has limited functionality, as smartphone screens are difficult to read, especially for diabetes patients with eye diseases (Andreas, aged 71 years; Katja, aged 43 years). Furthermore, all users, regardless of technological (or financial) restrictions, considered the lack of connectivity among different devices—smartphones, insulin pumps, and sensors—to be problematic (Conrad, aged 31 years; Emma, aged 45 years):

Every device does its own things.

Andreas, aged 71 years

To overcome this issue, Gerd, aged 32 years, who had a background in computer programming, even tried to create or at least adjust his own apps for better connectivity. Overall, we saw widespread use of mobile devices for self-testing, with some connected to participants’ smartphones, whereas others were not. This gave patients more autonomy in their lifestyle and health management, but technology reliability remained suboptimal, particularly with missing connectivity, low usability, and an even stronger reliance on smartphones, which were already used for a plethora of other everyday life activities.

The use of mobile devices, such as smartphones or fitness trackers, for lifestyle management is another aspect that we identified as a relevant use dimension. We observed 3 rather distinct appropriation styles, including nonusers who either considered the tracking of lifestyle information (eg, steps, calorie intake, and weight) to be more or less useless (Petra, aged 25 years; Ramona, aged 43 years) or used tracking, particularly step tracking, with an app (Daniela, aged 32 years) or a fitness tracker (Olga, aged 52 years; Marc, aged 64 years) but had ceased this usage. For others, such technology was a source of inspiration to become more active. Gerd, aged 32 years, used the location-based game *Ingress* to get more active. Ben, aged 64 years, was proud of his *AppleWatch* and its functionalities, an important symbolic aspect, and Emma, aged 45 years, integrated the *Polar App* and *Watch* into her daily routines. Lifestyle management with mobile devices was carried out by some of our participants mainly by focusing on step counters through apps, fitness trackers, or smartwatches. Nonetheless, a discrepancy existed between patients’ needs and app availability. Patients, particularly those with type 2 diabetes, saw great potential for apps to help with their eating habits (Emma, aged 45 years; Jessica, aged 56 years). Katja, aged 43 years, wanted to use a nutrition app but had not found any suitable choices. These diabetic patients were looking for an app to track their everyday life behaviors and compute the required insulin doses accordingly, thereby allowing them to act spontaneously and freely in their everyday lives.

#### Social Prestige and Control

The use of mobile devices and/or apps, particularly for testing, gave technologically savvy (type 1) patients a sense of agency. This was accompanied by an increase in social prestige, as they became experts not only with respect to their own disease but also with respect to new technologies (Fiona, aged 29 years; Gerd, aged 32 years; Olga, aged 52 years). First, meta-communication about diabetes and diabetes management comes into play as other patients recommended these technologically savvy individuals with diabetes to test technology or asked them about testing results. For example, Gerd, aged 32 years, a software programmer, was proud of his self-programming of apps to solve existing problems and fulfill other diabetic patients’ needs. Second, besides enhanced agency and the claim of being a pioneer, social prestige was enriched from a device perspective. For example, in the words of Ben, aged 64 years:

There is a kind of luxury [owning the newest devices].

In this way, Ben points to the issue of social status *vis-a-vis* appropriation.

## Discussion

### Principal Findings

Our objective was to provide a more comprehensive view of mHealth use for diabetes self-management beyond the simple question of adoption versus rejection. Study 1, conducted in Singapore, specifically aimed to identify evaluations of diabetes-app use and appropriation for self-management. Most importantly, study 1 revealed that diabetes patients do not merely use specific diabetes apps for their daily diabetes self-management but rather make use of their whole mobile-media ecosystem, such as other health apps, chat apps, or Web-based databases. Building on these findings, we broadened our perspective in study 2, conducted in Germany, more generally examining not only diabetes-app use but also mobile-media use in diabetes self-management, including information search and retrieval, and monitoring linked to diabetes self-management.

The semistructured interviews revealed several functional, normative, and restriction evaluations that play a role in both studies, whereas symbolic evaluations only appeared in study 2. In particular, the following functional, symbolic, normative, and restriction evaluations were identified (RQ1):

1. Functional evaluations mainly refer to the use of diabetes apps for diabetes monitoring, such as log-book apps used for recording and sharing results with health care providers and the use of diabetes apps for nutrition information. However, patients also mentioned using alternative mobile apps and channels for their daily diabetes self-management, namely apps for general health information (eg, fitness apps, *WebMD*), as well as apps to communicate with other patients (eg, *WhatsApp*).
2. Symbolic evaluations were not observed among Singaporean patients but appeared to play a role in the German study. The use of mobile devices and apps gave patients a feeling of agency and a boost in social prestige through owning a new device or through instilling a sense of being technological pioneers. However, Singaporean diabetes patients did not mention any of these aspects, which might be explained by sociocultural differences between the 2 countries: German society is conceived as more individualistic than collectivistic Asian societies [74]. Thus, using mHealth for diabetes self-management as a vehicle to demonstrate technical knowledge and social status might only play a role in more individualistic cultures.
3. In terms of normative evaluations, we found that patients’ relationship with their doctors plays a major role. This observation is linked to a second cultural difference in our 2 studies. Singaporean patients who were highly dependent on their doctors’ recommendations were hesitant to use diabetes apps, as their doctors did not actively advise using them, as they seemed reluctant to recommend apps that the Singaporean government had not sanctioned. In the context of sociocultural barriers proposed by the information communication technologies for health care model [60,61], the collectivist nature of the Singapore society is premised on Confucian principles [74] that might play a role here. As extant studies show, further reasons for doctors’ reluctance, including in Germany, could be a lack of perceived usefulness, technical concerns, and familiarity and privacy issues [75,76].
4. Finally, diabetes patients indicated several restriction evaluations, namely financial, temporal, cognitive, and technological barriers to diabetes-app use. Regarding technological barriers, diabetes patients complained about their smartphones’ small screen sizes, dependence on battery life, and apps’ incompatibility with older smartphones, other devices such as insulin pumps, and other patients’ needs.

As proposed by the MPA model in the context of mobile phone appropriation, meta-communication also plays a role in diabetes self-management with diabetes apps (RQ2). Interestingly, diabetes patients discuss topics around diabetes management mainly with other diabetes patients, in online diabetes support groups or via *WhatsApp*. In contrast, physicians only play a minor role, as they are reluctant to recommend or mention diabetes apps.

Regarding RQ3, study 2 revealed 3 overarching appropriation patterns emphasizing that the use and everyday-life integration of mHealth in diabetes self-management are not restricted to simple diabetes-app use:

1. The *mediated communication* pattern embraces information gathering about diabetes, including therapies and medication, using traditional forms of eHealth via computers and mobile devices, as well as connectedness and exchanges with peers and other diabetes patients face-to-face via chat functions or online support groups. Restrictions in this pattern mainly refer to mobile phones’ small screen sizes and a lack of trustworthy information on the Web.
2. The *diabetes self-management* pattern includes self-treatment and monitoring, as well as lifestyle-management. This pattern mirrors what is typically described as the use of mHealth for diabetes self-management, namely the use of smartphones to measure and track blood glucose levels and the use of self-management apps. In this context, the interviews revealed important restrictions, such as a feeling of dependence on smartphones (eg, battery life), compatibility issues tied to different devices and app versions, screen size (especially for patients with eye diseases), and costs. In addition, support on adequate nutrition and physical activity appeared to be a further dimension. However, in this context, use patterns are very diverse, and several patients stopped using such apps after a while, possibly because of discrepancies between patients’ needs and apps’ functionalities, as patients would prefer a one-size-fits-all app that fulfills all their needs.
3. The *social prestige and control* pattern refers to the symbolic aspect of mHealth use for diabetes. It mostly develops with experienced app use as it gives patients a feeling of empowerment regarding both their own disease situations and the use of new technologies. With more expensive devices, social prestige also comes into play. In this pattern, meta-communication plays a specific role, as experienced patients can serve as opinion leaders to support other less-experienced patients.

Thus, studies 1 and 2 complement each other in 3 aspects:

1. We expanded our research focus by focusing not only on specific diabetes apps (study 1) but also on the use of smartphones within the whole mobile-media ecosystem (study 2). In this paper, we see that mHealth and eHealth apps go hand in hand with mHealth, expanding traditional forms of eHealth, such as using the internet for information-gathering and (social) exchange. *WhatsApp*, as a mobile-specific communication app, plays an important role, as it helps connect patients with each other and acts as both an information source and a communication tool for fellow patients, friends, family, and, to a lesser extent, doctors. However, as already demonstrated in study 1, doctor-patient communication about diabetes apps, as well as meta-communication in general, is limited. Therefore, we reiterate earlier calls [18] for greater integration of technological innovation within the overall health care system rather than perceive them as stand-alone entities.
2. This expansion in our focus highlighted that differences between type 1 and type 2 patients were not limited to diabetes-app use but included the entire appropriation process of mobile technologies for diabetes self-management. It appears that type 1 patients, potentially because of their greater need to manage diabetes, were more technologically savvy. In addition, acquiring digital skills to use such technology became an important aspect for gaining control over their lives, as well as respect from fellow patients, friends, and colleagues. In this context, it should be noted that to gain a broad picture of mHealth appropriation in the diabetes context, we included both type 1 and type 2 diabetes patients in our studies, with the results indicating both commonalities and differences between these 2 patient groups. Among type 1 diabetic patients, mHealth use was more generally common; they also used food databases more often, probably because of these patients having a greater need to monitor food ingredients. This pattern evolved both in studies 1 and 2, indicating that self-treatment and self-testing (eg, blood glucose levels) were more relevant to type 1 patients, giving them more control and autonomy, whereas lifestyle management was relevant to both types. Apart from that, use and appropriation patterns with various mobile devices and apps were rather similar in both patient groups. As this study did not focus on detecting differences between type 1 and type 2 diabetes patients, future studies should examine the potential differences further.
3. We looked at diabetes self-management in 2 different health systems, that is, Singapore and Germany. Although we did not primarily intend to compare mHealth use in different health systems or countries, looking at 2 distinct contexts allowed us to look for similarities and differences: functional, normative, and restriction evaluations were very similar between Singapore and Germany, although sociocultural differences emerged. Regarding restriction evaluations, we noted monetary and technological restrictions as recurring patterns in both studies. Regardless of the health system, monetary costs played a crucial role, which were particularly relevant for type 1 patients. Furthermore, patients needed devices to be compatible with each other. In addition, we found, in both cases, that even older patients with diabetes were interested, at least partially, in new technology and successfully employed strategies to integrate mHealth devices and smartphone apps into their diabetes self-management.

### Limitations

Further research also is needed to address a few limitations. First, we conducted the 2 studies in 2 different countries despite the fact that we did not intend to compare appropriation processes. On the contrary, it was our objective to extend research that we conducted in Singapore both to a broader perspective on mHealth appropriation and to another country to assess recurring patterns in 2 different cases. The 2 countries are comparable regarding the relevance of diabetes, industrialization, smartphone penetration, and the presence of a well-developed health system. However, cultural differences must be acknowledged. This might explain why we did not find symbolic evaluations in Singapore, whereas in Germany, social prestige evolved as a relevant aspect of mHealth appropriation. It also should be noted that even if both countries rank among the top nations regarding technological development, it remains unclear how widely attitudes toward technology differ in Singapore versus Germany. Our results did not reveal any major differences regarding smartphone use for diabetes self-management; nevertheless, future research should consider this aspect in more detail. In addition, we did not systematically control for the influence of socioeconomic status and thus cannot make any statements on this. Finally, because of rather small sample sizes, qualitative research generally lacks generalizability in the results. However, a qualitative approach was a necessary step to gain deeper insights into diabetes patients’ appropriation patterns and everyday lives and to identify evaluations relevant to them [77]. In the next stage of research, our findings can be used to develop standardized questionnaires to gain insights into the distribution of appropriation patterns among a broader population.

### Conclusions

The study of mHealth apps for diabetes management is in a nascent stage with not only promising results but also many open questions [12-19]. In this project, studies 1 and 2 revealed that appropriation of mHealth for diabetes self-management is not limited to using specific diabetes apps but rather includes patients’ entire mobile-media ecosystem. Even if diabetes apps play a role, especially for self-treatment and self-testing, diabetes patients use many more digital resources when dealing with their conditions, such as lifestyle apps, messenger apps, traditional health-information websites, or forums accessed from a computer or mobile device. Thus, mHealth is important for diabetes self-management but in multiple ways that go far beyond diabetes-app use. In addition, our findings indicate that mHealth cannot substitute for interpersonal communication, for example, with other patients, peers, or health care providers, but it complements and supports interpersonal communication, especially via messenger apps. However, doctor-patient communication only plays a minor role in this context and can even be a barrier to mHealth use, as doctors are reluctant to recommend using mobile apps. Further reasons for diabetes patients to be reluctant to use mHealth for diabetes (continuously) include financial, technical, cognitive, and temporal issues. Apart from individual constraints (eg, eye problems, technical skills, and use of outdated devices), it appears that patients still cannot find what they are looking for. Patients want a not-yet-existent app that combines everyday-life requirements while computing correct insulin doses, thereby allowing for more freedom and spontaneity. Thus, considering these recurring evaluations, the appropriation of mHealth for diabetes self-management could be enhanced if doctors were less reluctant to recommend self-management apps, if connectivity between devices and apps, including multipurpose apps, was improved, if apps adapted to users’ physical restrictions (eg, font size), and if people already using mobile media for diabetes self-management were integrated into new apps’ development process and used as consultable opinion leaders to facilitate appropriation for others.

However, even if some overarching issues evolve, not every app solution can accommodate each and every patient. This is reflected in the 3 overarching dimensions of mHealth appropriation for diabetes self-management that we uncovered: (1) mediated communication (information-gathering, connectedness, exchange), (2) diabetes self-management in a narrow sense (self-treatment and monitoring, lifestyle-management), and (3) social prestige and control (symbolic aspects of mHealth use for diabetes). Diabetes patients differ highly in respect to these dimensions, that is, *how* they use mHealth for diabetes self-management, for *what purposes* they use it, and how they *evaluate* use. Integrating this knowledge into future mHealth apps’ designs and effect studies might shed more light on mHealth’s great potential for diabetes self-management.

## Abbreviations

CGM: Continuous Glucose Monitoring
eHealth: electronic health
mHealth: mobile health
MPA model: Mobile Phone Appropriation model
RQ: research question
TAM: Technology Acceptance Model
UTAUT: Unified Theory of Acceptance and Use of Technology

## Appendix

Multimedia Appendix 1 Interview guide (Singapore).

Multimedia Appendix 2 Interview guide (Germany), translated into English.
