# Supplementary material for: Appropriation of Mobile Health for Diabetes Self-Management: Lessons From Two Qualitative Studies
Source: JMIR Diabetes. 2019 Mar 29;4(1):e10271. doi: 10.2196/10271 (PMC6460309; doi:10.2196/10271)
Supplement: Multimedia Appendix 1 [file diabetes_v4i1e10271_app1.pdf]

Interview guide – Singapore

Diabetes condition and treatment plan

- Please, tell me a little bit about your diabetes condition, your treatment, and your daily diabetes routine.
- Who is supervising you in your diabetes care? Who takes part in your disease management?
- Who usually makes the decisions for your treatment procedures?
- Do you prefer to make your own decisions regarding diabetes treatment or to follow your supervisors' instructions?
- Do your supervisors adapt treatment to your wishes and needs?
- Do your HCPs usually take time to explain things in detail to you?
- How much do you feel you are autonomous and independent in dealing with your diabetes?

A considerable part of treatment is self-management of the disease.

- Please explain a little bit how important your diabetes care & self-management is for you.
- In which way do you think your self-management could improve?
- What, do you feel, would you really need in your current self-management?
- How much, do you feel, can you influence your diabetes outcomes?
- Do you feel, you can influence your BG values/other values with your self-management?
- How much control, do you feel, do you have over your diabetes?
- About your own self-management:
    a. Do you follow a diet/eating plan?
    b. Do you exercise?
    c. Do you test your BG values?
    d. Do you take care of your feet?
    e. (Do you smoke?)
- How did it feel, when you were diagnosed for the first time? Were you scared? How did your feelings change over time? Did you feel depressed at some point?
- How confident do you feel about your own self-care now? Do you feel you have the competence for a good self-management?
- In what ways does/did your family/your friends support you or were/are involved in your self-management?

Diabetes app use and perceived utility

- What type of smart device do you usually use (private use)?
- Did you ever use anything on a smart device that was related to your diabetes management? Like a blood glucose diary app or something similar.
- Yes:
    a. Please explain a little bit how you use(d) your smart device for self-management.
    b. What were your experiences with diabetes management through the

smart device? Was it helpful? Why?

- No:
    a. Are there any reasons why you didn't use your smart device(s) for diabetes management before?
    b. Are you generally interested in using your smart device for diabetes management?

Let's look at one diabetes app/tool together.

The interviewee is shown the study smart phone with the selected app (MySugr) if he/she never used a diabetes app, or is asked to open an app on the private phone that he/she uses/d. The app (background) is briefly explained if the app is unknown (for the ones who never used an app). The interviewer clicks through the app and the provided features showing them to the interviewee (or the interviewee clicks through them him-/herself). The interviewee is asked to comment on the usefulness of these features for him-/herself. The app usability test should last max. 5 minutes.

Diabetes app use and perceived utility

- How important would the following features in apps be for you/ how much would the following features influence your decision to use an app (long- term)? (5-point scale from 1= "not important at all/very low influence on app use" to 5= "very important/very high influence on app use", scale* is shown to the participant) à explain if necessary
    a. Features to directly communicate with your HCP (doctor/nurse/dietician)
    b. Features that support self-management activities (e.g. BG measurement)
    c. Features to stay in touch with other users regarding the self-management, e.g. family, or friends, or other patients
    d. Features that make the app specifically relevant to you (adapted to your needs)
    e. Features that support your feeling of competence for self-management
    f. Features that support your choices in self-management and treatment
    g. Features showing you the impact of your self-management on your disease outcome
- What would you expect from a smart device diabetes tool to meet your needs for self-management? What online or offline tools would you prefer to use?
- What does it depend on, if you use an app for longer/what would it depend on to make you use an app for longer? à explain if necessary
- Do you talk about smart device options for supporting self-management with any of your diabetes supervisors?
- What would you think about getting an app prescribed that is used in cooperation with your doctor?
- How much do you think the following aspects influence your decision to start using a diabetes app and/or to use a diabetes app for longer: (5-point scale from

1= “very low influence on app use” to 5= “very high influence on app use”, scale* is shown to the participant) à explain if necessary

a. The relationship to your HCP (support of the app use by him/her, e.g. doctor, nurse, dietician)
b. The communication with your HCP about the app use
c. Your other self-management behaviors (diet, exercise, BG measurement)
d. The support by others regarding the app use (e.g. friends and family, other patients)
e. The relevance of a specific app for you
f. Your feeling about your own app usage competence (technology)
g. Your own initiative and initiation of the app use (that the use is not forced)
h. The impact you feel the app use has for your disease management

Patient empowerment and apps

- Have you heard of empowerment of patients to manage a disease more independently and with own responsibility?
  Explain understanding of empowerment briefly (definition**)
- Do you think smart devices/apps could promote empowerment? What is the role of smart devices in diabetes management in your opinion?
- Do you think tools on smart devices can improve self-management of diabetes patients?

To summarize:

- Do you think diabetes patients should rather be independent and manage their disease themselves with supervision of their doctor, or should the doctors rather manage the disease and the patients should just follow the doctor’s instructions? Why?

Additional optional questions

- Do you use alternative means to manage your disease (than consultations with your supervisors)?
- Where do you see barriers for yourself in using a diabetes app in your future self-management?
