# Supplementary material for: Appropriation of Mobile Health for Diabetes Self-Management: Lessons From Two Qualitative Studies
Source: JMIR Diabetes. 2019 Mar 29;4(1):e10271. doi: 10.2196/10271 (PMC6460309; doi:10.2196/10271)
Supplement: Multimedia Appendix 2 [file diabetes_v4i1e10271_app2.pdf]

Interview guide – Germany

Everyday life & disease

First of all, I'd like to get to know you a little better.

- Please tell me a little bit about your diabetes disease and treatment.
    - Year of diagnosis?
    - Treatment since when? (participation in screening programms)
    - Who treats / cares for them? (requency of contact, contact person)
    - Which kind of treatment? (Insulin, injections vs. oral therapy)
    - Do you participate in further education / health courses?
    - If you think of a typical day, what does it look like? (daily routines in relation to the disease)
- Let's talk about your treatment now, who can have a say in it?
    - Who do you trust to treat you?
        - Your own decisions?
        - Treatment plan of the doctor / diabetes assistant?
        - Advice of friends?
    - To what extent do the caregivers adapt their treatment to your wishes and needs?
        - Do your caregivers take time to explain things to you?
        - Do you feel free to decide how to treat your disease?
- How did you feel when you were diagnosed with diabetes?
    - Emotions: Fear, anger, despair, helplessness, resignation
        - Other patients told us, that they felt dejected and sometimes depressed. How did you feel?
        - How have feelings changed over time?

Usage of smartphones & wearables

Thank you very much for your openness. Now we come to something completely different. As I said before, I am interested in your use of a smartphone or a wearable.

- Please tell me a little bit about your smartphone.
    - Since when do you use it? Which device?
- When you pick up your smartphone: What do you do first? (e.g., WhatsApp, Facebook, checking mails)
    - Can you tell us, what you use your smartphone for?
    - If not mentioned: Do you use social networks, like Facebook, Instagram, or Snapchat?
        - If not used: Why do you not use social networks?
        - If not mentioned: Do you use messenger apps, like WhatsApp?
        - If not used: Why don’t you use messenger apps, like WhatsApp?
- How do you know, how to use your smartphone?
    - Do you ask others for advice, if you have questions about using your smartphone?
- Do you use any wearable, e.g., activity tracker or smartwatch?
    - Since when?
    - Which device?
- When you pick up your ### (insert): What do you do first?
    - For example: check daily steps, calorie consumption

    - What do you use your ### (use) for?
    - If not mentioned: Control of movement (steps), calorie consumption, ...?
- How do you know, how to use your ### (insert)?
    - Ask others for advice, if you have questions about using your ###?
- If I feel bad, I use my smartphone to take my minds off other things. Do you know that, too? (here: emotional skills)
    - Are there any situations, in which you consciously reach for the smartphone or consciously put it aside?

Self management & self-efficacy & empowerment

- Treating diabetes also means that patients with a health-conscious lifestyle can influence the course of the disease. To what extent, do you think, can you influence the course of the disease yourself?
    - Could you influence the blood sugar level yourself?
        - YES: how and why?
        - NO: why not?
    - How much control, do you think, you have over your condition?
    - How important is controlling your disease to you?
- How secure do you feel about dealing with your disease now?
- To what extent does your family or friends support you in dealing with diabetes or how do you involve them?
    - Where would you like more support?
- Do you exchange information about the disease with other patients?
    - Online or offline?
    - In what form?
    - How do you know the people affected?
- Have you ever informed yourself about the course of diabetes on your smartphone?
    - If yes: How did you proceed?
    - If not: Please imagine that you would like to find out about the course of diabetes on your smartphone. How would you proceed?
- Do you know certain apps or applications that can help you deal with diabetes? (knowledge)
    - examples: forums, self-help groups (for advice and actual support), WhatsApp support with regards to the disease in general or with regards to specific questions
    - What applications are these?
    - Where do you know these applications from?
- Have you used your smartphone/### in connection with your diabetes, e.g. to count steps or to monitor blood sugar levels? (Application)
    - YES: What do you use your smartphone/your ### for?
        - What are your experiences?
        - Useless? Why?
        - Useful? Why?
    - NO: Are there any reasons, why you haven't used your smartphone/your ### (use) so far for this purpose?
        - Are you generally interested in using your smartphone/your ### (use) in connection with the disease?
- At what point could you imagine (further) support from your smartphone/your ###

(use)?
    - Do you think that smartphones or ### (use) can promote, prevent or improve the handling of the disease? Why?
    - In addition, where do you come up against limitations in dealing with the disease where the smartphone/the ### (use) cannot help you? Why? Whom do you contact then?
- I have a Smartwatch that is connected to an iPhone and tells me, when to move more. However, sometimes I consciously decide against it and stay seated. (Adapt devices to own property)
    - How about you? Do you sometimes decide against the advice of your smartphone / your ### (insert)?

Future

- Now we're almost at the end of the interview. I'm finally interested in, whether you are a person who enjoys new things, who also tries something out, or would you rather wait and see what others experience with new developments and then decide for or against? (technology affinity)
- If you could express your wishes for something: How do you think you could improve your diabetes management? (related to yourself)
    - Is there anything that could improve the support of your diabetes management? (related to other supporters)

End

Now we have come to the end of the interview. Thank you very much for your time. Is there anything else we forgot?
